# The efficacy of one-stage laparoscopic versus two-stage endo-laparoscopic management of cholecystocholedocholithiasis

**DOI:** 10.1186/s12893-025-03094-2

**Published:** 2025-07-30

**Authors:** Medhat Anwar, Shaimaa Abdelaziz Abdelmoneim, Mahmoud Hamida, Mohamed Samir, Mohamed Abu Deeba, Magdy Hassan, Mohamed Hany, Bart Torensma, Mohamed Hefzy

**Affiliations:** 1 Medical Research Institute, Alexandria University, Alexandria, Egypt; 2 Clinical Research Administration, Alexandria Directorate of Health Affairs, Egyptian Ministry of Health and Population, Alexandria, Egypt; 3 Clinical Epidemiology, Erasmus MC, Rotterdam, the Netherlands; 4 Department of Surgery, Medical Research Institute, Alexandria University, 165 Horreya Avenue, Hadara, Alexandria 21561 Egypt

**Keywords:** Cholecystocholedocholithiasis, Laparoscopic, One-stage, Cost-effectiveness, Endoscopic, Surgery

## Abstract

**Background:**

Over the past 20 years, one-stage laparoscopic common bile duct exploration (LCBDE) combined with laparoscopic cholecystectomy (LC) has gained wide acceptance for the management of cholecystocholedocholithiasis (CCL). Despite this, the two-stage endo-laparoscopic approach, consisting of endoscopic retrograde cholangiopancreatography (ERCP) followed by LC, remains the most commonly used strategy. This study aims to analyze the efficacy of one-stage laparoscopic management versus two-stage endo-laparoscopic management of CCL.

**Methods:**

This study included 100 patients with CCL, with data collected retrospectively for those admitted between January 2018 and December 2020, and prospectively between January 2021 and June 2021 at the Surgical Department of the Medical Research Institute Hospital, University of Alexandria. Patients were divided into two groups: Group A, who underwent two-stage management (ERCP followed by LC), and Group B, who underwent one-stage management (LCBDE and LC). Outcomes compared included procedural success, duration, hospital stay, and cost.

**Results:**

In our economic analysis, the one-stage laparoscopic strategy demonstrated substantially lower costs ($3,636) compared to the two-stage approach ($5,682), representing a savings of $2,046 per patient. Procedural failure and conversion to open surgery occurred in 4% of Group B patients (2 cases) compared to 8% of Group A patients (4 cases). The median duration of the one-stage procedure was longer at 155 min compared to 95 min for the two-stage procedure. Hospital stay was comparable, with a median of 3 days in both groups. Readmissions were rare, with no cases in the one-stage group and one case in the two-stage group.

**Conclusions:**

The one-stage laparoscopic approach for managing CBD stones and gallstones offers substantial cost savings compared to the two-stage approach ($2,046 per patient). This approach presents a viable option for healthcare systems that prioritize resource efficiency.

## Background

Common bile duct (CBD) stones are frequently managed using pre-, intra-, or post-operative endoscopic retrograde cholangiopancreatography (ERCP). However, laparoscopic common bile duct exploration (LCBDE) has gained substantial acceptance over the past two decades [1–3]. In Egypt, the prevailing approach to managing CBD stones involves a two-stage procedure, typically consisting of ERCP followed by laparoscopic cholecystectomy. Different teams often perform these stages in separate locations, a practice that significantly impacts costs, complications, and hospital stay.

Evidence indicates that CBD stone clearance success rates for LCBDE range from 75 to 100%, compared to 62–96% for ERCP. One-stage interventions, where LCBDE is performed concurrently with laparoscopic cholecystectomy, offer several advantages, including a reduction in the number of procedures, lower overall costs, and shorter hospital stays [2–5].

Therefore, this study compares clinical success and cost analysis of a one-stage versus two-stage approach to managing CBD stones.

## Methods

This study included patients diagnosed with concomitant common bile duct (CBD) stones and gallstones, who were admitted to the Surgical Department at the Medical Research Institute Hospital, University of Alexandria. The study was conducted in two phases: a retrospective phase, covering admissions between January 2018 and January 2021, and a prospective phase, spanning from January 2021 to June 2021. Written informed consent was obtained from participants. The study conformed to the principles of the Declaration of Helsinki and was approved by the ethical committee of the Medical Research Institute, University of Alexandria, under number/438/dec/2020. Clinical trial number: not applicable.

### Two-and-one stage procedures

Two distinct management groups were created on the treatment approach they had received: *Group A* comprised patients who underwent a two-stage procedure consisting of endoscopic retrograde cholangiopancreatography (ERCP) followed by laparoscopic cholecystectomy (LC) on separate days, while *Group B* included those who received a one-stage procedure comprising laparoscopic common bile duct exploration (LCBDE) and LC during a single operative session. This fundamental difference in timing—separate occasions versus same surgical sitting—represented the core distinction between the management approaches analyzed in this retrospective review. All procedures had been performed by the surgical team according to the standard institutional protocols for both pre-operative assessment and post-operative care.

### Inclusion criteria

The study included male and female patients diagnosed with concomitant common bile duct (CBD) stones and gallstones (CCL), which were confirmed through ultrasonography, computed tomography (CT), or magnetic resonance cholangiopancreatography (MRCP). These patients were subjected to either the standard two-stage endo-laparoscopic procedure, consisting of endoscopic retrograde cholangiopancreatography (ERCP) followed by laparoscopic cholecystectomy (LC), or a one-stage intervention, which involved laparoscopic common bile duct exploration (LCBDE)—either trans-cystic or trans-ductal—combined with laparoscopic cholecystectomy (LC). Size and number of common bile duct (CBD) stones–>10 mm and multiple stones were in favor for one-stage. CBD diameter– A dilated CBD (> 10 mm) often favored a one-stage approach (Lap. CBD exploration (LCBDE) + LC) due to the technical feasibility of intraoperative clearance.

### Exclusion criteria

Patients who were admitted for urgent endoscopic retrograde cholangiopancreatography (ERCP) due to acute cholangitis were excluded from the study. Additionally, cases involving recurrent common bile duct (CBD) stones, pancreatic duct stones, or pancreatic divisum were not included. Patients with a history of biliary diversion surgery or significant medical comorbidities such as chronic renal disease (CRD), chronic liver insufficiency (CLI), or ischemic heart disease (IHD) were also excluded. Furthermore, individuals classified as American Society of Anesthesiologists (ASA) class 4 or 5, as well as pregnant patients, were not eligible for participation in the study [6].

### Assessment criteria and outcome measures

The two groups were evaluated based on several key variables. The duration of each procedure was recorded in minutes, with the total time for the two-stage management group being the combined duration of the endoscopic retrograde cholangiopancreatography (ERCP) and laparoscopic cholecystectomy (LC). The outcome of each procedure was classified as either a success or a failure. Additional variables assessed included the rate of readmission, the length of hospital stay (in days), and postoperative outcomes. For the two-stage group, the hospital stay for ERCP was added to the stay following LC.

Postoperative outcomes were evaluated using the Clavien-Dindo classification [7] system at three distinct time points: immediately postoperative, at 30 days, and at one year. Cost analysis was conducted using the 2021 Egyptian Unified Procurement Authority (UPA) list for consumables and therapeutic supplies, including drugs, intravenous fluids, anesthetics, guide wires, cannulas, sphincterotomes, laparoscopic clips, syringes, tubes, catheters, balloons, stents, and other necessary items. Additionally, basic hospital costs—such as hospital stay, operating room expenses, durable instrument usage, and staff costs—were calculated based on the 2021 Egyptian Health Insurance Authority list, assuming that patients were admitted to a second-class room. All costs were then converted to United States dollars for the year 2024 using the CCEMG-EPPI-Centre Cost Converter.

### Statistical analysis

Descriptive and inferential statistics were used. All data were first tested for normality using the Kolmogorov–Smirnov test, Q–Q plot, and Levene’s test. Categorical variables were expressed as n (%). Continuous normally distributed variables were represented as mean and standard deviation, and non-normally distributed data were represented as the median and interquartile range (IQR) for skewed distributions. To compare categorical variables among different groups, we used Pearson’s chi-square test or Fisher’s exact test when appropriate. Normally distributed continuous data were tested with independent samples, and the student’s t-test was used to compare continuous variables between independent samples. For skewed data, the Mann–Whitney U test was used.

## Results

The demographic data revealed a predominance of female patients in both groups, with a stronger tendency in Group A. The mean ± SD age of patients in Group A was 43.60 ± 16.02, with 72% being female, compared to Group B, where the mean ± SD age was 53.76 ± 15.45, and 54% were female (Table 1).

**Table 1:** Comparison between the two studied groups regarding age and sex

	Group A(*n* = 50)		Group B(*n* = 50)		*p*
	No.	%	No.	%	
Sex					
Male	14	28.0	23	46.0	0.062
Female	36	72.0	27	54.0	
Age (years)					
Mean ± SD.	43.60 ± 16.02		53.76 ± 15.45		0.002^*^

*SD* Standard deviation test, *p* *p* value for comparing between the studied group

The one-stage procedure’s median duration was longer than the two-stage procedure’s, with a median of 155 min (120–300) for the one-stage approach versus 95 min 40–190 for the two-stage approach (40 min for ERCP and 55 min for LC). Despite this difference in procedure time, there was no significant difference in hospital stay between the two groups, with both having a median stay of 3 days (2–8) and 3 (2–18) (Table 2).

**Table 2:** Distribution of the studied cases according to hospital stay and duration of the procedures

	Group A			Group B	*p*
	**ERCP**	**LC**	**Total**		
Hospital stays (Day)					
Median (min-max)	1.0 (1.0-1.0)	2.0 (1.0-7.0)	3.0 (2.0-8.0)	3.0 (2.0-18.0)	0.900
Duration (Minute)					
Median (min-max)	40.0 (40-40)	55.0 (40-150)	95.0 (40-190)	155.0 (120-300)	<0.001^*^

*p* p value for comparing between Total group (A) and Group (B)

*Statistically significant at *p* ≤ 0.05

A total of five patients in Group B (one-stage approach) had undergone prior ERCP before being treated with LC + LCBDE. Four patients had failed stone extraction during ERCP and were subsequently managed with the one-stage approach, and one patient had failed cannulation during ERCP, necessitating a one-stage surgical approach as an alternative treatment.

These cases were included in the one-stage group as they ultimately underwent LC with LCBDE as the definitive intervention.

In terms of clinical outcomes, the one-stage strategy demonstrated a marginally higher success rate (96% vs. 92%) compared to the two-stage strategy. The conversion rate to open surgery was also lower in the one-stage group (4% vs. 8%). These differences may reflect the older average age in the one-stage group (54 years vs. 47 years) as well as possible confounding factors such as previous attacks of inflammation, adhesions, and the learning curve associated with LCBDE, which is a relatively newer technique compared to ERCP and LC.

Complications were assessed at three postoperative time points: immediately after surgery, at 30 days, and at one year. There were no significant differences in the complication rates between the one-stage and two-stage procedures at any time point (Table 3).

**Table 3:** Comparison between the two studied procedure according to complications

CD Grade	Postoperative period		30- day period		One year period	
	G (A)	G (B)	G (A)	G (B)	G (A)	G (B)
(-)	33	30	45	46	50	48
GI	16	20	5	3		
GIIIb	1					
N/A				1		2

*CD* Clavien-Dindo, *G* Grade, *(-)* No deviation from normal postoperative course

A cost-analysis was performed to compare the expenses of the two-stage procedure ($5,682) with the one-stage procedure ($3,636). However, this cost-minimization finding requires further studies that incorporate quality of life outcomes.

## Discussion

The present study aimed to compare clinical outcomes of the one-stage (LCBDE + LC) and two-stage (ERCP + LC) strategies for the management of concomitant common bile duct (CBD) stones and gallstones. This study contributes to the growing body of evidence favoring the one-stage approach, particularly in terms of operational efficiency and cost, despite the slightly longer procedural duration.

The demographic analysis revealed a higher prevalence of gallbladder disease among premenopausal women, which accounts for the gender disparity observed in our study. In postmenopausal women, the incidence of gallbladder disease appears to converge with that of men, reflecting a narrower gender gap. This finding is consistent with existing literature that links hormonal factors to gallbladder disease prevalence in women.

The one-stage procedure’s median duration was longer than the two-stage procedure’s, with a median of 155 min (120–300) for the one-stage approach versus 95 min 40–190 for the two-stage approach (40 min for ERCP and 55 min for LC). However, this result is only partially surprising. Several randomized trials and meta-analyses have reported longer operative times for the one-stage approach [2–5]. The increased time for LCBDE + LC can be attributed to the complexity of bile duct exploration, particularly in patients with failed preoperative ERCP, adhesions from previous inflammatory episodes, and the inherent challenges posed by the procedure’s learning curve. Despite this longer operative time, the median hospital stay was the same for both groups (3 days), although the mean hospital stay was shorter for the one-stage group (3.9 days vs. 4.5 days). This finding supports previous studies, which have demonstrated reduced hospital stays in patients undergoing the one-stage procedure [2, 3, 5, 8–10].

The cost analysis showed that the one-stage procedure ($3,636) was less expensive than the two-stage procedure ($5,682). Still, these results should be taken with caution because further research is needed to include quality of life factors in the cost evaluation.

Prior to this study, evidence from randomized controlled trials and meta-analyses had already established the safety and efficacy of the one-stage strategy for managing CBD stones. Success rates for the one-stage procedure have been reported to range between 75% and 96.8%, with postoperative morbidity rates as low as 3.6% [5, 8, 9]. In comparison, the two-stage strategy has shown success rates between 61.7% and 94.6%, with a higher associated postoperative morbidity ranging from 5.1 to 29.8% [8]. These studies, like ours, have demonstrated that while the one-stage approach may be more technically demanding and time-consuming, it offers significant advantages in terms of cost, reduced morbidity, and shorter hospital stays.

The challenge of managing CBD stones has traditionally centered on balancing the risks and benefits of endoscopic versus surgical interventions. ERCP followed by LC has long been considered the standard of care, particularly in institutions where expertise in LCBDE is limited. However, as laparoscopic techniques have improved and are widespread, the advantages of combining CBD exploration with cholecystectomy in a single procedure are becoming increasingly clear.

The success of this approach relies heavily on the surgical team’s expertise, which may limit its widespread adoption, particularly in resource-limited settings. Efforts should be made to provide targeted training and skill development for surgeons in LCBDE to facilitate broader implementation of this approach.

### Limitations

Several limitations of this study must be acknowledged. First, the study’s retrospective design for a portion of the data introduces potential biases, such as selection and recall, which may affect the internal validity of the findings. Retrospective studies often rely on medical records and missing or incomplete data can skew results. A fully prospective design would provide stronger evidence and allow for more rigorous control of confounding variables.

Second, the sample size in this study was relatively small, which may limit the generalizability of the findings. Epidemiologically, this restricts the ability to detect smaller, but potentially important, differences between the two management strategies. Larger, multi-center trials with a more diverse patient population would be needed to validate these results and increase external validity.

Additionally, the study did not fully account for potential confounding factors such as differences in patients’ baseline characteristics, including comorbidities or previous history of CBD stone interventions, which could influence outcomes like postoperative recovery or quality of life. In epidemiological studies, controlling for these confounders is crucial to avoid misinterpretation of associations between interventions and outcomes.

Another important limitation is the relatively short follow-up period; a longer follow-up period is necessary to understand better the durability of the health benefits associated with either strategy.

## Conclusion

The one-stage laparoscopic approach for managing CBD stones and gallstones offers substantial cost savings compared to the two-stage approach ($2,046 per patient). This approach presents a viable option for healthcare systems that prioritize resource efficiency.

## Data Availability

The datasets used and/or analysed during the current study are available from the corresponding author on reasonable request.

## Abbreviations

CBD: Common bile duct
LCBDE: Laparoscopic common bile duct exploration
LC: Laparoscopic cholecystectomy
CCL: Cholecystocholedocholithiasis
ERCP: Endoscopic retrograde cholangiopancreatography
CT: Computed tomography
MRCP: Magnetic resonance cholangiopancreatography
ASA: American Society of Anesthesiologists
IQR: Interquartile range

## References

[1] Dasari BV, Tan CJ, Gurusamy KS, Martin DJ, Kirk G, McKie L et al. Surgical versus endoscopic treatment of bile duct stones. In: The Cochrane Collaboration, editor. Cochrane Database of Systematic Reviews. Chichester, UK: John Wiley & Sons, Ltd; 2013. p. CD003327.pub3. 10.1002/14651858.CD003327.pub3 23999986

[2] Ramser B, Coleoglou Centeno A, Ferre A, Thomas S, Brooke M, Pieracci F, et al. Laparoscopic common bile duct exploration is an effective, safe, and less-costly method of treating choledocholithiasis. Surg Endosc. 2024;38:6076–82. 39138682 10.1007/s00464-024-11139-5

[3] Nie S, Fu S, Fang K. Comparison of one-stage treatment versus two-stage treatment for the management of patients with common bile duct stones: A meta-analysis. Front Surg. 2023;10:1124955. 36816010 10.3389/fsurg.2023.1124955 PMC9935819

[4] Rogers SJ, Cello JP, Horn JK, Siperstein AE, Schecter WP, Campbell AR et al. Prospective randomized trial of LC + LCBDE vs ERCP/S + LC for common bile duct stone disease. Arch Surg. 2010;145. 10.1001/archsurg.2009.226 20083751

[5] Bansal VK, Misra MC, Rajan K, Kilambi R, Kumar S, Krishna A, et al. Single-stage laparoscopic common bile duct exploration and cholecystectomy versus two-stage endoscopic stone extraction followed by laparoscopic cholecystectomy for patients with concomitant gallbladder stones and common bile duct stones: a randomized controlled trial. Surg Endosc. 2014;28:875–85. 24162138 10.1007/s00464-013-3237-4

[6] Statement. on asa physical status classification system. 2020.

[7] Clavien PA, Barkun J, De Oliveira ML, Vauthey JN, Dindo D, Schulick RD, et al. The Clavien-Dindo classification of surgical complications: five-year experience. Ann Surg. 2009;250:187–96. 19638912 10.1097/SLA.0b013e3181b13ca2

[8] Noble H, Tranter S, Chesworth T, Norton S, Thompson MA, Randomized. Clinical trial to compare endoscopic sphincterotomy and subsequent laparoscopic cholecystectomy with primary laparoscopic bile duct exploration during cholecystectomy in higher risk patients with choledocholithiasis. J Laparoendosc Adv Surg Tech. 2009;19:713–20. 10.1089/lap.2008.0428 19792866

[9] Ding G, Cai W, Qin M. Single-stage vs. two-stage management for concomitant gallstones and common bile duct stones: a prospective randomized trial with long-term follow-up. J Gastrointest Surg. 2014;18:947–51. 24493296 10.1007/s11605-014-2467-7

[10] Zhu J, Wang G, Xie B, Jiang Z, Xiao W, Li Y. Minimally invasive management of concomitant gallstones and common bile duct stones: an updated network meta-analysis of randomized controlled trials. Surg Endosc. 2023;37:1683–93. 36278995 10.1007/s00464-022-09723-8

